# The Role of Maxillofacial Structure and Malocclusion on Condylar Displacement in Maximum Intercuspation and Centric Relation in Patients Seeking Orthodontic Treatment—A Scoping Review

**DOI:** 10.3390/jcm12020689

**Published:** 2023-01-15

**Authors:** Ilona Radej, Ewelina Dargiewicz, Magdalena Sawczuk-Siemieniuk, Raúl Ferrando Cascales, Álvaro Ferrando Cascales, Rubén Agustín-Panadero, Izabela Szarmach

**Affiliations:** 1 Department of Orthodontics, Medical University of Białystok, ul. Waszyngtona 15A, 15-274 Białystok, Poland; 2 Faculty of Health Sciences, San Antonio de Murcia Catholic University (UCAM), 30107 Murcia, Spain; 3 Prosthodontic and Occlusion Unit, Department of Stomatology, Faculty of Medicine and Dentistry, University of Valencia, 46010 Valencia, Spain

**Keywords:** centric relation, centric dental occlusion, mandibular condyle, dental articulators, orthodontics, dental cast analysis, cephalometrics

## Abstract

Background: Available knowledge about malocclusion and cephalometric variables and their connection with an increased risk of condylar displacement (CD) is scarce. This article aims to present current information on the relationship between centric relation-maximum intercuspal position discrepancies and maxillofacial morphology and malocclusion in patients seeking orthodontic treatment as well as to identify those who require expanded diagnostic evaluation for this disorder. Methods: This review analyzed the PubMed, Cochrane Library, Web of Science, and Scopus electronic databases up to February 2022. Keywords and additional manual searches were performed. Literature selection was based the PRISMA-ScR checklist. The JBI Critical Appraisal Tool assessed the methodological quality of included studies. Results: The databases search provided 2321 studies. A total of 10 studies were included in this review after eligibility criteria and JBI assessment. This review was separated into five parts that evaluated CD correlations depending on the following: maxillofacial structure in different vertical and sagittal skeletal patterns, vertical, horizontal, and transverse malocclusions. Conclusions: A hyperdivergent facial skeletal structure is a risk factor for increased CD, particularly in the vertical dimension. The condylar processes are usually displaced in a posteroinferior direction. Further studies are warranted to elucidate the relationship among remaining skeletal and dental malocclusions and the occurrence of CD.

## 1. Introduction

Centric relation (CR) is defined as the unstrained, physiologic, maxillomandibular relationship. This is the most anterosuperior position of the mandibular heads against their articular surfaces. The repeatability of this reference position of the mandible makes it clinically useful [1]. Okeson characterizes this as the most musculoskeletally stable position of the mandible [2]. CR has become the focus of treatment for many orthodontists, chiefly because of Roth’s extensive work. In the 1970s, a series of articles appeared dealing with the positions of the condyles and their importance in orthodontic therapy. A novel concept at the time, functional orthodontics was added to the orthodontic treatment process which had previously focused on facial esthetics, dental esthetics, healthy periodontium, and treatment stability. It was suggested to use an articulator when planning orthodontic treatment [3,4,5]. Articulator-mounted casts may be helpful in certain cases for the orthodontist to better picture the occlusal conditions in a stable musculoskeletal position such as the CR, especially in cases of large occlusal discrepancies [2]. The CR is a sought-after physiologic position also in orthodontic treatment as shown by many professionals within the field of the gnathic system and its functioning [6,7,8,9,10,11,12]; however, not all authors are in agreement regarding this approach. Zonnenberg et al. regard that the position determined by the MIP should be viewed as clinically acceptable. They reason that that every individual has a unique temporomandibular joint (TMJ) relationship which cannot be described by any singular term [13].

The presence of occlusal interferences may cause the muscular system to react in two ways. The first results in condylar displacement (CD) from CR to the maximum intercuspal position (MIP). The second pathway may result in physical contact with posterior teeth and the presence of an anterior open bite whereby the CD is less pronounced [14]. The majority of CDs are clinically undetectable. For this reason, identification and quantification of these displacements with instrumentation and articulator mounts in a seated condylar position are recommended for a proper orthodontic diagnosis in certain cases. Diagnostic accuracy may be increased by eliminating muscle splinting [15,16,17]. However, there is also disagreement among authors which necessitates further studies and discussions on this subject [18,19].

The etiology of TMD is viewed as multifactorial. Possible etiologies include malocclusions, varied cranial morphology, physical trauma, stress, individual predispositions, and structural conditions [10]. Recent scientific trends have focused increasingly on biophysical etiologies [20,21]. Condyle position may play a role in the etiopathogenesis of temporomandibular disorders (TMD) as one of many factors [22]. Data from certain studies back the notion of increased CD with occlusal instability as well as dysfunction [23,24,25,26,27]. In previous studies, a clinically significant CD threshold of 2 mm in the vertical and horizontal planes was considered diagnostic and 0.5 mm in the transverse plane may have adverse effects on the TMJ [7,8,27,28]. In some TMD categories, the condyles are seen to be positioned more posterosuperomedially. This position may result from a change in the position of the articular disc [29]. Additionally, transverse CD, frequently presenting with dental cross bite, may also be linked with TMD [24]. However, it should be kept in mind that instrumental analyses and assessment of CD cannot be used for the diagnosis of TMD in isolation from the clinical exam. The cause-and-effect relationship between occlusion and TMD is based on the anatomical observation between tooth position and jaw function and on the higher incidence of TMD in individuals with malocclusion compared to the general population [30,31,32,33]. Although a strong association between TMD and malocclusion has not been found, several features of malocclusion, such as open bite, deep bite, posterior crossbite, and larger interincisal angle, have been suggested [34]. Of particular note are skeletal class II profiles and hyperdivergent growth patterns, which are more probably associated with an increased occurrence of displacement of the TMJ disc and degenerative disorders. However, the cited systematic review is not conclusive as to these observations [35]. Nonetheless, evidence from large population studies shows weak and inconsistent associations, suggesting that the role of occlusion in the etiology of TMD should not be overemphasized [36]. The concept of treatment in the CR as a preventative measure in order to improve the relationship between the disc and condyle has not been verified in studies [37,38]. Kandasamy et al. suggested current concepts of biopsychosocial assessment and management be used in patients with TMDs rather than mechanistic models in approaching dental and skeletal malalignments [39]. Since there is no proven causal relationship, the role of malocclusion in the etiology of craniomandibular disorders should be considered as limited. Patient awareness and concerns relating to their occlusal conditions must be taken seriously by clinicians during general and dental examinations, as maladaptive behaviors may be iatrogenic in nature. Routine examination related to TMD before commencement of orthodontic treatment seems to be crucial [36,40]. Orthodontists often treat patients during puberty, and the symptomatology of TMD may not be as obvious at this stage, which is why identifying patients who may develop joint disorders is so important. Further research on this topic is warranted due to such differing opinions.

In order for the clinician to be able to estimate the risk of increased CD, it is desirable to conduct research into the dependence of CD on the maxillofacial structure. Paknahad et al. assessed the skeletal relationship of CD by cone beam computed tomography (CBCT). Their work showed that CD correlated significantly with sagittal and vertical skeletal craniofacial morphology [41,42]. Park et al. similarly noted that the condylar position and morphology vary according to vertical facial morphology [43]. Patients with hyperdivergent skeletal patterns have a tendency to present more superiorly positioned condyles when compared with hypodivergent skeletal patterns [43,44]. Moreover, some studies indicate a connection between hyperdivergence and TMD. [45,46] Other authors, in turn, noted a relationship between internal derangement with dolichofacial skeletal patterns and an increase in mandibular plane angles [47,48]. Contrastingly, Hidaka et al. [49] noted no relationship among face type and the position of the condylar process. Similarly, Aboalnaga et al. [50] provided strong evidence for a reduced relationship between TMD and malocclusion. Such differing findings and conclusions again justify further research in this area.

The use of the mandibular position indicator (MPI) or condylar position indicator (CPI) is recommended to determine the position of the condylar process. It should be noted that the presence of a slide or shift at the level of the occlusion may not accurately characterize the three-dimensional transformation of the position of the condylar axis [7,49,51,52,53,54,55,56,57,58]. This is because significant translation of the hinge axis may occur due to rotation of the mandible without obvious movement in the anterior region of the dentition [59]. Only a small part of the horizontal component of the CR-MIP slide, as seen at the incisal level, is due to horizontal translational displacement of the condyles [14]. The change in occlusion at the dental level is a complex consequence of the bilateral spatial displacement of the condyles. The condyles do not move symmetrically, and they do not sit at the same level with the dentition. Therefore, it is difficult to predict the CD through measurement of occlusal changes [58]. With this in mind, changes at the level of the occlusion and the condylar process should be assessed separately.

There is no definitive review of studies that has assessed malocclusions and cephalometric variables and their connection with an increased risk of CD. This makes it challenging to ascertain which skeletal types and dental anomalies should be clinically assessed along with mounting models. This study aims to review available literature on CD, taking into account maxillofacial morphology and malocclusion. Such a review may help identify which patients particularly require an extended diagnosis and to draw the attention of orthodontists to this group of patients when planning orthodontic treatment.

## 2. Materials and Methods

### 2.1. Protocol and Registration

This scoping review was based on the Preferred Reporting Items for Systematic Reviews and Meta-Analysis extension for Scoping Review (PRISMA-ScR) statement protocol. The PICO (population, intervention, comparison, and outcomes) strategy was: population: orthodontic patients; intervention: MPI, CPI, mounting models; comparison: between patients with different maxillofacial morphologies and different types of malocclusions; outcomes: CD in MIP and CR.

### 2.2. Data Sources and Search Strategy

On 14 February 2022, two independent researchers (IR, ED) performed a search of the target online databases for eligible studies. PubMed (with MEDLINE), Web of Science, Cochrane Library, and Scopus online records were searched in order to identify suitable articles. Search strategy and terms used for the search are shown in Appendix A. No filters were applied during the search. A backward citation chaining of eligible full-text studies was performed.

### 2.3. Eligibility Criteria

Table 1 outlines inclusion and exclusion criteria.

### 2.4. Search Results

**Figure 1:**
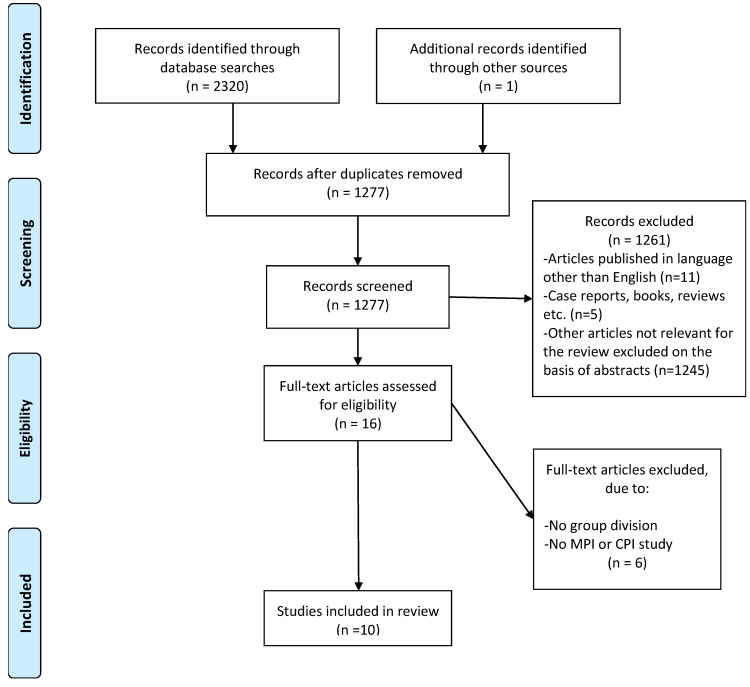
Preferred Reporting Items for Systematic Reviews and Meta-Analysis (PRISMA) search strategy flowchart. MPI = mandibular position indicator; CPI = condylar position indicator.

The initial database examination yielded 2321 results. EndNote X9 software (Clarivate Analytics, Philadelphia, PA, USA) filtered out any duplicates and a manual removal was then performed, leaving 1277 search results. Sixteen papers were preliminarily selected for systematic review after an analysis of abstracts was applied and inclusion and exclusion criteria were taken into account. Full texts were ultimately analyzed by two independent researchers and ultimately 10 original publications were selected for this review. The systematic review search and selection process according to PRISMA is presented as a flowchart in Figure 1.

### 2.5. Critical Appraisal of Individual Sources of Evidence

One original paper included in this review was described as a cross-sectional study by the authors. The remaining 9 papers were defined as observational/descriptive studies. The quality of the included studies was assessed according to the Joanna Briggs Institute (JBI) Critical Appraisal Checklist For Studies Reporting Prevalence Data [60]. Two independent investigators assessed a given study’s research evidence according to a 9-question form, pertaining to study design, conduct, and reliability of results (yes, no, unclear, and non- applicable). Researchers discussed any discrepancies that arose in order to come to a consensus. The following elements were particularly taken into account—study group selection and allocation, research methods, and statistical analyses.

### 2.6. Data Analysis

All results were analyzed descriptively. Qualitative and quantitative data from the articles included study type, country of origin, sample size, sex distribution, age distribution, type of skeletal pattern and/or malocclusion, distribution of the number of subjects in groups, presence of TMD symptoms, the method of registering and deprogramming CR, evaluated variables of CD in the anteroposterior (delta x), vertical (delta z), and transverse (delta y) axes.

## 3. Results

Seven of the ten studies included in this review assessed CD size according to maxillofacial structure [14,49,51,61,62,63,64]. Skeletal facial type was assessed by a lateral cephalogram and measured cephalometric variables. The cephalogram was taken in the MIP [14,61,62,64]. The remaining studies did not specify the conditions for taking the x-ray images. Additionally, the seven studies included in the review assessed the size of CD according to malocclusion [49,51,58,63,64,65,66]. These studies were published in the time span from 1995 to 2022 and included patients from various countries such as India [61], USA [51,62], Japan [49], Spain [63], Portugal [14], Poland [64], Turkey [65,66], and China [58]. Sample sizes fluctuated from 48 [64] to 162 [63] patients. The summary of the quality assessment of the reviewed articles is in Table 2. Table 3 summarizes descriptive characteristics of the included publications. The mean values of Δx, Δy, and Δz displacement, taking into account the division of patients into groups, is presented in Table 4. Table 5 and Table 6 present the number of extreme displacements in the TMJ and displacements of >1 mm, depending on the availability of these data in the research work.

### 3.1. Assessment of CD According to Maxillofacial Structure in Different Vertical Skeletal Patterns

Vertical cephalometric measurements were taken into account in six of the admitted studies [14,49,61,62,64]. CD values were compared in the vertical (Δz) and horizontal (Δx) planes, and in three studies, the transversal (Δy) was also measured [49,61,64]. A study by Ponces et al. compared CD measurements in three types of faces in the population [14]. The study included 36 patients in each of the facial pattern groups as follows: hyperdivergent, hypodivergent, and intermediate. Vertical displacement was more pronounced than horizontal displacement in every group. Vertical displacement differed between hyperdivergent and hypodivergent patients and between hyperdivergent and intermediate patients significantly. CD was more common in the group of hyperdivergent patients. Condyles were dislocated laterally more frequently in hyperdivergent and intermediate groups. In the hypodivergent group, they were displaced anteriorly.

Girardot [62] and Chandra et al. [61] conducted studies that focused on comparing CD in patients with hyperdivergent and hypodivergent face types. Girardot studied 66 patients, 33 of each type. Similarily, Chandra et al. studied 70 patients with 35 in each group. In these studies, the hyperdivergent subjects had greater CDs in both horizontal and vertical dimensions. In the study by Chandra et al., the condyles were deflected rearward 3.6 times greater in the hyperdivergent group than in the hypodivergent group.

Radej et al. assessed the effects of craniofacial structure and occlusal conditions on the positions of the articular heads of the mandibular condyles [64]. After analyzing 48 patients, no statistically significant correlations of Δx and Δz displacements with vertical cephalometric measurements on a lateral cephalometric radiograph (SN-ML, SGo/NMe) were observed by the authors. They did find a correlation between Δy and the mandibular plane angle, which may have been due to modified masticatory muscle function in those with an abnormal vertical face shape. The condyles were displaced mainly inferoanteriorly (58.3% patients), which was likely due to the studied population’s prevalent deeper facial structures.

Hidaka et al. [49] studied 150 patients, having divided them into groups, noteably according to mandibular plane angle, and assessed differences in condylar position between CR and MIP. Significant differences in CPI measurements were not observed between the groups. The condyles were displaced most commonly inferiorly with a smaller anteroinferior component.

### 3.2. Assessment of CD According to Maxillofacial Structure in Sagittal Skeletal Patterns

Skeletal classes according to ANB were assessed in three studies [51,63,64]. Both Radej et al. [64] and Utt et al. [51] did not find any statistically significant correlations between CD and ANB angular measurements based upon a group of 48 and 107 patients, respectively. The aim of a large study conducted by Barrera-Mora et al. [63] was to assess the correlation between malocclusion, benign joint hypermobility syndrome, condylar position, and TMJ symptoms. Among the many valuable conclusions based on the studied group of 162 patients, they noted statistically significant correlations between vertical discrepancies of centric sliding (Δh) and skeletal classes. They observed an increase of Δh in skeletal class I compared with skeletal class II.

### 3.3. Assessment of CD According to Vertical Malocclusions

Vertical malocclusions were assessed in two of the admitted studies. Ari-Demirkaya et al. [65] not take into account cephalometric variables in their research. They divided 90 patients into three groups of 30: normal overbite, deep bite, and open bite. The authors observed that open bite cases had a great CD in the vertical plane. The percentages of individuals presenting with a vertical CD > 1 mm was significantly higher in the open bite group (50%) than in the deep bite (27%) and control groups (17%) (*p* = 0.017). Displacement from CR to MIP showed great variability in terms of directions. Their study also presented that open bite cases present with shorter protrusion paths compared to normal and overbite cases. Additionally, a transverse slide > 1 mm was seen more often in the deep bite group (33%) than in the other two groups (13% each) (*p* = 0.082). Barrera-Mora et al. [63] did not find a correlation between CD and a dental malocclusion pattern. The dental malocclusion assessment in the latter study included, among others, open bite malocclusions.

### 3.4. Assessment of CD According to Horizontal Malocclusions

Horizontal malocclusions were analyzed in six of the admitted studies. He et al. [58] studied a group of 50 patients, among which 25 had a diagnosed Angle’s class I molar position and the remaining 25 had an Angle’s class II. They observed that the vertical displacement on both sides was greater than the anteroposterior displacement, with a significant difference on the right side. No significant difference was found between Angle class I and II patients and the magnitude of the CD. In sagittal directions, the condyles displaced primarily posteriorly and inferiorly. In a study by Utt et al. [51], no significant difference was also observed between Angle class I and II groups of patients upon comparing MPI measurements [51]. None of the factors studied in this study enabled the clinician to identify which patients had a significant CR-MIP discrepancy. Similarly, Barrera-Mora et al. [63] did not find a statistically significant correlation between Angle’s class and CD.

Radej et al. [64], however, found a relation between Angle’s classification and anterioposterior CD values. They observed that in Angle’s class I patients, the condyles were displaced anteriorly (median Δx = 0.3 mm). This explained the presence of Angle’s class II in models recorded in the CR, which upon achieving first contact in class II, may then slide anteriorly and ultimately make contact in MI in class I. In Angle class II cases, the median displacement of Δx = −0.3 indicated that the condyles were positioned more rearwards, which after mounting the models in the CR, may then present the dental defect as less severe. However, this is not obvious because the displacement at the level of occlusion does not strictly correspond to the displacement at the level of the condylar process.

Among patients studied by Hidaka et al. [49], Angle’s class I, II, III were assigned to 44, 84, and 22 individuals, respectively. A significant 0.2 mm displacement towards the left side (*p* < 0.01) was noted by the authors in the patients with Angle class III in comparison with class I and II, in which mean displacement was near zero. This characteristic of the class III group may be, at least partly, connected to the finding that excessive mandibular growth is often asymmetric and higher frequency of asymmetry is often found in class III patients in comparison to other groups.

Turasi et al. [66] compared groups of patients with large and normal overjet. Each group consisted of 33 patients. The authors found that CD, both vertically and transversely, was significantly larger in the overjet group (*p* = 0.030 and *p* = 0.008). The number of individuals presenting with a CD larger than 1 mm as well as the range of CD in the anterioposterior direction was larger in the overjet than in the control group. This was statistically significant solely for the right side (*p* = 0.045).

### 3.5. Assessment of CD According to Transverse Malocclusions

Transverse malocclusions with CD were analyzed in three of the admitted studies. The authors Radej et al. [64] showed a correlation between mandibular midline shift with asymetrical horizontal displacement of the condylar process. This study showed that a downwards CD was commonly greater on the left side, similarly to Hidaka et al.’s publication [49]. The left condyle shifted anteriorly in the maximum intercuspal position (median shift 0.25 mm), meanwhile the right condyle barely moved (median shift 0.03 mm). Such unevenness caused a mandibular midline shift to the right in the MIP. Statistical analyses showed these findings to be significant (*p* = 0.029). Hidaka et al. likewise noted marked CD asymmetry, observing a greater downwards displacement on the left side while a forward movement was greater on the right. These characteristics may result in the mandible’s anterior portion to be displaced leftward. Hidaka et al., however, noted only a weak positive correlation in this direction.

In turn, Barrera-Mora [63] et al. showed a correlation between Δy and transversal malocclusion. Increased Δy values were observed in patients with an anterior crossbite in comparison with an increased overjet. Radej et al.’s study did not confirm this last observation, however, the number of patients with crossbite was low and such an observation may have been difficult to note [64].

## 4. Discussion

Understanding the correlation between the craniofacial structure and malocclusion and the mandibular condyles’ positions may help practitioners in their orthodontic treatment in the diagnosis and planning stages, when the initial position of the condyles often varies. Precise condylar position in the CR in three spatial axes may be assessed by the MPI or CPI. If a CD is present, this should be taken into account, so as not to exacerbate the already present displacement. Particular attention is required in cases of extractions as studies have shown signs of posterior condyle repositioning after treatment [67]. The clinical significance of such displacement is unproven and requires further investigation, nevertheless, the lack of evidence should not decrease the clinician’s vigilance. Similar attention should be paid to the use of elastics during orthodontic treatment, especially for Class II patients, which increases the stress on the TMJ. It is still unclear whether this may predispose to signs and symptoms and TMD, but clinicians should be aware of that possibility [68]. Furthermore, significant differences are present in the occlusion when it is influenced by the teeth and when it is influenced by the condyles. It has been observed that models mounted in the CR position show the following dental interarch characteristics when compared with the dental interarch relationship seen intraorally or from models hand-articulated in an MIP: premature contacts posteriorly (94.0%), enlarged overjet, reduced overbite, midline changes, and Angle classification changes [7,28,53,54]. Such observations may affect the direction and extent of planned orthodontic tooth movement. Orthodontic treatment changes the patient’s occlusive conditions and abnormal tooth contact is one of the potential risk factors for the development of TMD. As such, orthodontists should provide the patients with orthopedically stable occlusal conditions that minimize the risk of TMD [2]. One should be aware that creating a “perfect” bite as an attempt to treat or prevent TMD, regardless of the type of braces used, is not supported by scientific evidence [69]

### 4.1. CD in Different Vertical Skeletal Patterns and Malocclusions

In all the presented studies assessing vertical disturbances, vertical displacements of the condyles were greater and more frequent in relation to displacements in the horizontal plane, which was also noticeable when extreme displacements were compared [14,49,61,62,64,65]. Greater vertical displacement is expected in both high-angle and low-angle patients. The frequency of these displacements varied considerably, which possibly were a result of varied inclusion criteria, anatomical differences in the TMJ structure, or insufficient deprogramming of patients.

In most of the above studies, vertical displacement was observed more frequently to a greater degree in high-angle patients than in low-angle patients. Only Hidaka et al. [49] did not find any significant differences between the groups. Studies by Chandra et al. [61] and Girardot [62] showed larger average Δx and Δz in hyperdivergent than in hypodivergent patients. Displacements in this group were more frequent in both planes. These differences were especially pronounced when comparing condylar processes displaced by 2 mm or more. Similar relationships were observed in the study by Radej et al. in patients with posterorotation which corresponded to the hyperdivergent face type, compared to patients with anterorotation [64]. Additionally, a study by Ponces et al. [14] showed higher and more frequent CD in the vertical plane in the group of hyperdivergent patients compared to hypodivergent or intermediate patients. Similarly, open bite can be treated as a hyperdivergency, as shown by a study by Ari-Demirkaya et al. [65], even though the study did not take into account cephalometric variables. The study observed a larger vertical CD in open bite patients compared to patients with deep bite [65]. The increased occurrence of CD in the vertical plane in patients with an open bite was explained by the posterior primary contact acting as fulcrum, which is much easily established if there is no incisal guidance in the front. Movement of the condylar processes from the first point of contact to the MIP is then usually a vertical rather than a horizontal movement. Hyperdivergent patients often have impaired incisal guidance and this argument seems to be a logical explanation for this entire group of patients. The geometry of the skeletal pattern, and particularly the mandible, may result in small changes of condylar position giving profound effects at the level of the dentition [62]. Based on the above studies, an orthodontist can predict a greater CD in patients with a hyperdivergent face type, especially in the vertical dimension. This observation confirms the theory that hyperdivergent patients present with a higher risk of TMD development [70].

However, the above studies are not completely in agreement in terms of the correlation of vertical cephalometric variables with the magnitude of CD in the horizontal plane. In most studies, patients with vertical disturbances in the direction of the open bite showed greater or more frequent displacements in the horizontal plane [61,62,64,65]. However, in a study by Ponces et al. [14] assessing both groups in the horizontal plane, the results were different than in the previous studies. Greater displacement was observed in hypodivergent patients, although this result was not statistically significant.

The situation is even more complicated in regard to the direction of horizontal displacements. In studies by Ponces et al. [14], Chandra et al. [61], and Girardot [62], the most common extreme displacements were noted to be directed posteriorly in hyperdivergent groups, which confirm earlier reports [8,27,53]. Girardot’s [62] study showed that condyles were generally displaced a larger distance in the hyperdivergent group in the anterior direction compared to the hypodevergent group. However, Ponces et al. [14] noticed more frequent extreme anterior displacements in hypodivergent individuals. Forward movement of the condyle may be due to premature contacts leading the mandible in this direction. These differences can also be caused by the different patterns in which the muscles work in different types of faces. In hypodivergent structures, the elevator muscles are aligned vertically across the first molars and are stronger whilst being positioned more forward as compared to hyperdivergent patients, which leads to more force applied anteriorly. A study by Radej et al. [64], where the condyles were mostly located anteroinferiorly (58.3%), confirms this finding. Similarly, in a study by Chandra et al. [61], anterior movement was greater in the group of hypodivergent patients, although this result was not statistically significant. A study by Ari-Demirkaya et al. [65] did not show any significant differences in the direction of the condyle in the sagittal plane between the groups. Assessment of the condyle position by CBCT also showed a great variety in hypodivergent patients and the position of the condyle in hyperdivergent patients being more predictable [70].

The study by Radej et al. [64] additionally draws attention to the correlation between the transverse displacement of the condylar process and the mandibular plane inclination angle, showing greater displacement in patients with posterorotation. Ari-Demirkaya et al. [65], however, noted larger lateral slides in deep bite cases. There seems to be no logical explanation for this. Therefore, when diagnosing patients with abnormal vertical skeletal pattern and malocclusion, particular care should be paid to not only vertical and horizontal displacements, but also transverse displacements. This may be due to abnormal muscle function in this type of face. As for vertical disturbances, the remaining studies showed no differences between the groups in transverse displacements of the condyle [14,49,61,62].

### 4.2. CD in Different Sagittal Skeletal Patterns and Malocclusions

The majority of the studies agree that skeletal class II malocclusions have greater anteriorly and superiorly positioned condylar processes in the articular joint space than the general population on CBCT scans. Upper or upper and posterior joint space narrowing was the most common deviation in skeletal class III cases [70]. Afzal et al. [71] showed significant difference in values of ANB in MIP and CR based on lateral cephalograms. According to the authors, special attention should be paid to patients with Angle’s class III for the centric slide. The studies included in the review, however, did not confirm these observations and did not show a significant correlation of the ANB angle with CD in the three spatial planes [51,63,64]. The authors Barrera-Moora et al. [63] only noticed an increase in Δh in skeletal class I patients as compared to skeletal class II. This value is the height difference in the incisal pin from CR and MIP. Therefore, we should not disregard patients with correct sagittal skeletal relationships during the diagnostic stages.

Half of the studies also showed no dependence of the CD on Angle’s classification [51,58,63]. Radej et al. [64] noted the presence of Angle class II on models recorded in CR in patients who were originally diagnosed as Angle class I, which is a common phenomenon in orthodontic practice. This observation resulted from an increased anteroposterior CD in this group of patients. Often, Angle class I does not seem to be a complicated malocclusion to treat, however, the diagnosis of a patient in CR may change the treatment mechanics and anchorage needs of the case.

It is widely accepted that MIP in the majority of individuals is located 1–2 mm away from CR in the anteroposterior direction [51,62,65,72]. Ackerman and Proffit [73] recommended to use the point of initial contact in assessing the occlusion when a shift greater than 1 to 2 mm between MIP and the point of initial tooth contact in CR closure is present. Therefore, irregularities that exceed 2 mm in the horizontal or vertical axes are of particular importance in diagnostics. Properly conducted orthodontic treatment may reduce the discrepancy of CR-MIP [74]. A study performed by Turasi et al. [66] agreed that a CR-MIP discrepancy less than 2 mm may be viewed as normal, as this was the situation for most of the subjects in the large overjet as well as the control groups whilst keeping in mind that no subjects presented with any TMD signs or symptoms. The authors noted greater vertical and transverse CD in the overjet group, which may have been due to the pathological stretching of the articular structures due to the increased overjet. CD examination in patients with increased overjet is justified in order to diagnose patients with CD > 2 mm and draw the clinician’s attention to the status of the TMJ of such a patient.

Hidaka et al. [49] observed significant transversal CD in Angle class III patients. This feature of class III malocclusions may be partly associated with the finding that excessive mandibular growth is often observed to be asymmetric and a higher frequency of asymmetry is seen in class III patients than other groups [75,76]. Additionally, the anatomical presentation of the articular fossa and eminence in dental and skeletal class III malocclusions potentially increase the risk of dislocation of the TMJ articular disc, thus these patients require particular care [70]

### 4.3. CD in Asymmetric Disorders

The MPI can be particularly helpful in diagnosing asymmetric malocclusion, which may be caused by condylar process displacement in the transverse plane or asymmetric displacement in the anteroposterior plane and/or vertical plane. Functional analysis of condylar movement undertaken by Ishizaki showed a close relationship between the direction of mandibular lateral displacement and the direction of condylar lateral shift during opening and closing [77]. Nevertheless, it should be remembered that displacement of the mandibular condyle may be only partly responsible for mandibular asymmetry. A more frequent occurrence of lateral displacement may also be associated with anatomical differences of the TMJ structures and/or dental arch forms [49]. Model analyses in CR may alter the original diagnosis made in MIP and alter the treatment plan as the asymmetry in CR will be less severe. Particular attention should be paid to patients with a mandibular midline shift and transverse malocclusions, such as crossbite.

A major limitation of this review is the small number of studies and the inherent heterogeneity among the included studies, in terms of both skeletal and dental defects. This review was limited to orthodontic patients, making it inappropriate to apply the results to the general population. The size of the groups was also very diverse and the division of patients into groups sometimes prevented the inclusion of a given group in the analysis due to the insufficient number of patients. Most studies analyzed asymptomatic patients [14,58,61,62,65,66]. The remaining studies enrolled patients with symptoms of TMD or this information was not available. In patients with TMD, CD may be increased and, therefore, the results of these studies may not be comparable. The majority of studies included adolescents having completed growth maturity and adults, but two studies also included children [49,51]. Children with incomplete development are characterized by a different structure of the TMJ, so including this age group together with adults may lead to incorrect conclusions. Nevertheless, this review is the first to summarize current available knowledge on this subject.

The quality of evidence in the studies included in this review was relatively low, with no control group established in half of the studies. Despite the lack of a control group, the studies were included in this review due to the small number of studies of this type. Nevertheless, the results of this review should be approached with caution. More high-quality research is required to estimate the increased risk of CD in specific skeletal or dental defects. Mounting models may be of great benefit malocclusions in which CDs occur more frequently. This would lessen errors in treatment planning and aid in the orthodontic diagnostic process. We suggest orthodontic mounting models as an extension of the orthodontic process.

## 5. Conclusions

The clinician can expect a larger CD in hyperdivergent facial patterns than in hypodivergent ones in both vertical and horizontal directions. Vertical displacements of the condyles in this group of patients are greater and occur more often in relation to displacements in the horizontal plane. The condyles are usually displaced posteroinferiorly. On the other hand, in hypodivergent patients, forward displacement can be expected, although the results of the studies are varied and there is insufficient evidence on this topic.

The varied nature and methodology of the studies included in this review makes it quite difficult to confidently establish a relationship between CD and the prevalence of most skeletal or dental malocclusions. Establishing such relationships requires further research and rigorous scientific methodology. Longitudinal studies are needed to identify CD as a possible risk factor for TMD.

## Figures and Tables

**Table 1:** Inclusion and exclusion criteria.

Inclusion Criteria	Exclusion Criteria
Papers evaluating effects of orthodontic variables on condylar displacement using a mandibular or condylar position indicator: (i) Cephalometric variables (ii) Malocclusion	(i) Not original articles (ii) Articles published in languages other than English (iii) Articles not conforming with PICO (iiii) Case reports, books, reviews

**Table 2:** JBL checklist for prevalence studies.

	Ponces et al. [14]	Chandra et al. [61]	Girardot [62]	Radej et al. [64]	Hidaka et al. [49]	Ari-Demirkaya et al. [65]	Barrera-Mora et al. [63]	Utt et al. [51]	He et al. [58]	Turasi et al. [66]
Was the sample frame appropriate to address the target population?	Y	Y	Y	Y	U	U	Y	U	Y	U
Were study participants sampled in an appropriate way?	Y	Y	Y	Y	Y	U	Y	Y	Y	U
Was the sample size adequate?	Y	Y	Y	U	Y	Y	Y	Y	U	Y
Were the study subjects and the setting described in detail?	Y	Y	Y	Y	Y	Y	Y	Y	Y	Y
Was the data analysis conducted with sufficient coverage of the identified sample?	Y	Y	Y	U	Y	Y	Y	Y	Y	Y
Were valid methods used for the identification of the condition?	Y	Y	Y	Y	Y	Y	Y	Y	Y	Y
Was the condition measured in a standard, reliable way for all participants?	Y	Y	Y	Y	Y	Y	Y	Y	Y	Y
Was there appropriate statistical analysis?	Y	Y	Y	Y	Y	Y	Y	U	Y	Y
Was the response rate adequate, and if not, was the low response rate managed appropriately?	Y	Y	Y	Y	Y	Y	Y	Y	Y	Y

Y—Yes; U—Unclear.

**Table 3:** Summary of the descriptive characteristics of included articles.

Author	Aim of Study	Country of Origin	Age (y.o)	TMD Symptoms	Number of Assessed patients/Joints	Main Group of Division	Assessed Variable CD	Method of Registering CR	Method of Deprogramming	Main Correlations and Conclusions
Ponces et al. [14]	Clarify the relationship between facial type and condylar position.	Portugal	12–46.2 (adolescents and adults)	asymptomatic	108/216 (36/72 per group)	hyperdivergent, hypodivergent and intermediate skeletal facial type	x, z	Roth’s power centric	10-min interposition of cotton roles	More extensive CD in the hyperdivergent group in the vertical dimension than hypodivergent and intermediate *. More extensive CD in the hypodivergent group in the horizontal dimension. No significant difference in horizontal displacement between groups.
Chandra et al. [61]	Evaluate and compare the CR and centric occlusion (CO) in asymptomatic subjects with hyperdivergent and hypodivergent facial skeletal type.	India	16–30 (young adult having completed growth)	asymptomatic	70/140 (35/70 per group)	hyperdivergent and hypodivergent skeletal facial type	x, y, z	modified Roth’s power centric	10-min interposition of cotton roles	Hyperdivergent subjects had greater displacement of the condyle in the horizontal dimensions *. Hyperdivergent subjects had greater rearward movement of the condyle *. Hyperdivergent subjects had greater displacement of the condyle in the vertical plane *. No differences between the groups in the medio-lateral dimension.
Girardot [62]	Determine if the CR to intercuspal position discrepancy was larger in patients with hyperdivergent facial type compared with patients with hypodivergent facial type.	USA	13–36 (young adult having completed or nearly completed growth)	asymptomatic	66 (33 per group)	hyperdivergent and hypodivergent skeletal facial type	x, z	modified Roth’s power centric	n/a	There was a statistically significant greater distraction of the condyles in the hyperdivergent group in both the horizontal and vertical planes.
Radej et al. [64]	To evaluate the impact of the craniofacial structure and occlusal conditions on the position of the mandibular condyles’ articular heads in the MIP and compare the CR and MIP of the mandibular condyles prior to orthodontic treatment.	Poland	11.5–50.3	asymptomatic and symptomatic	48/96	1. Helkimo Di0 and Di1 or Helkimo DiII or DiIII 2. Skeletal class I, II, III (ANB angle) 3. Hyper, normal and hypodivergent (SGo/NMe) 4. Posterior, normal and anteriorotation (SN/ML) 5. Angle’s classification I, II, II(1/2 unit), III, III(1/2) unit 6. Cross-bite and lingual occlusion or normal occlusion	x, y, z	Roth’s power centric	pulsatile biting of wooden spatula for a 10–15 min	1. No correlation of CD with cephalometric measurements (ANB, SN-ML, SGo/NMe). 2. Correlation between CD in the transverse axis and SN-ML angle *. 3. Correlation between CD in anteroposterior axis and a midline shift of the mandible *. 4. Correlation between Angle’s classification of molar position on the right side and anteroposterior CD values *. Cephalometric measurements (ANB, Sgo/NMe, and SN-ML) do not provide sufficient information to predict the frequency, size, and direction of CD at the level of the condylar processes. Cast analysis in an articulator is particularly desirable in patients with Angle class I, in whom an anterior CD may mask the occurrence of an Angle class II in CR. It would allow an assessment of whether the malocclusion is the result of an eccentric shift of the mandible, in which the asymmetrical displacement of the condyles results in a mandibular midline shift.
Hidaka et al. [49]	Report the frequency, magnitude, and laterality of differences in condylar position between CO and CR before orthodontic treatment. Investigate how the differences vary according to age, gender, mandibular plane angle, or Angle classification. Compare the results with those of the Caucasian race.	Japan	6.7–57.8	n/a	150	age, gender, SN to mandibular plane angle, Angle classification	x, y, z	Roth’s power centric	n/a	No significant differences in the magnitude of CPI measurements were found among any set of groups. Angle class III subjects tended to have significant condylar displacement toward the left side *. The frequency, magnitude, or direction of CO-CR changes at the level of the condyles cannot be predicted by age, gender, Angle classification, ANB angle, or mandibular plane angle.
Ari-Demirkaya et al. [65]	Compare normal overbite, deep bite, and open bite cases clinically healthy TMJ regarding the difference between condylar positions in CR and CO, condylar paths, and radiographic findings of condylar appearance in order to establish normative data.	Turkey	18–32 (adults)	asymptomatic	90/180 (30/60 per group)	Normal overbite, deep bite, and open bite	x, y, z	bilateral manipulation technique described by Dawson	10-min interposition of cotton roles	Open bite cases show larger vertical CR–CO slides and shorter protrusion paths than normal and deep overbite cases. The clinician should pay special attention to the TMJ status of open bite patients.
Barrera-Mora et al. [63]	To determine the association between TMD symptoms and condylar position, dento-skeletal malocclusion pattern, and benign joint hypermobility syndrome.	Spain	20–30	asymptomatic and symptomatic	162 (140 + 22 control group)	gender, degree of hypermobility joint, joint dysfunction parameters of the TMJ, malocclusion pattern at dento-skeletal level: skeletal class (I, II, III), malocclusions (normal occlusion, class I, II, III, open bite), sagittal malocclusion (normal overjet, anterior crossbite, increased overjet)	x, y, z, h	Roth’s power centric	n/a	No statistically significant relationship between benign joint hypermobility syndrome and the amount of condylar displacement or TMD, but such a relationship does exist with malocclusion patterns, especially, malocclusion Class II and open bite. Anterior crossbite and condylar displacements in the vertical plane are risk factors in developing TMJ symptoms.
Utt et al. [51]	Make a three-dimensional comparison of condylar position in CO relative to the clinically captured CR on patients before initiation of orthodontic treatment. Report the frequency and magnitude of the differences between CO and CR positions at the level of the condyles, and examine the relationship of condylar position changes to other factors routinely available and traditionally considered by orthodontists before treatment.	USA	7.75–38.17	n/a	107	Angle classification, ANB angular measurement, age, gender	x, y, z	Roth’s power centric	n/a	Patient age, ANB angle, gender, or Angle classification cannot be used to predict frequency, magnitude, or direction of CO-CR changes at the level of the condyles.
He et al. [58]	Study the changes of condyle position and occlusion between CR and MI positions.	China	14.5–18.5	asymptomatic	50 (25 per group)	Angle class I Angle class II	x, y, z	Roth’s power centric	n/a	When the mandible moved from CR to MI, overbite deepened, overjet decreased, and molar relationship became mesialized. No significant difference between Angle Class I and Angle Class II patients was observed in condylar position and occlusion changes.
Turasi et al. [66]	Compare normal overjet versus large overjet cases with clinically healthy temporomandibular joints; to establish normative data regarding the difference between condylar positions in CO and maximum intercuspation and deflective CO contacts.	Turkey	18–29	asymptomatic	66 (33 per group)	large and normal overjet group	x, y, z	bilateral manipulation technique described by Dawson	10-min interposition of cotton roles	Patients with increased overjet show some significant differences in the range and direction of CR-CO slides compared with normal overjet patients, even in healthy TMD-free cases. This study indicates that the clinician should pay special attention to the TMJ status of patients with a large overjet.

* statistically significant; delta x = condylar displacement in anteroposterior axis; delta z = condylar displacement in vertical axis; delta y = condylar displacement in transverse axis.

**Table 4:** Mean values of delta x, delta y, and delta z with division of patients into groups. (in mm).

Author	Mean Delta x	Mean Delta z	Mean Delta y	Predominant Direction of Movement
Ponces et al. [14]	0.84 hyperdivergent group 0.94 hypodivergent group	1.65 * hyperdivergent group 1.05 hypodivergent group	n/a	posterior in the hyperdivergent and intermediate groups, anterior in the hypodivergent group
Chandra et al. [61]	1.13 * hyperdivergent group (0.63 forward movement) (1.53 backward movement) 0.65 * hypodivergent group (0.95 forward movement) 0.44 backward movement)	1.46 * hyperdivergent group 0.99 * hypodivergent group	0.38 hyperdivergent group 0.39 hypodivergent group	n/a
Girardot [62]	1.21 * hyperdivergent group (37.25 total forward movement) (42.5 total backward movement) 0.66 hypodivergent group (17.5 total forward movement) (26.3 total backward movement)	1.7 * hyperdivergent group 1.2 hypodivergent group	n/a	n/a
Radej et al. [64]	0.15: 0.18 anterorotation group 0.06 normal rotation group 0.64 posterorotation group 0.35 Angle class I right −0.26 * Angle class II right 0.31 Angle class III right	0.76: 0.58 anteriorotation group 0.75 normal rotation group 1.32 posterorotation group 0.65 Angle class I right 0.79 Angle class II right 0.96 Angle class III right	−0.01: −0.13 anterorotation group 0.00 normal rotation group 0.26 * posterorotation group 0.04 Angle class I right −0.08 Angle class II right −0.07 Angle class III right	anteroinferior
Hidaka et al. [49]	−0.1 left 0.2 right	1.0 left * 0.9 right *	0.1 Angle class I 0.0 Angle class II −0.2 * Angle class III	downward with a smaller antero-posterior component
Ari-Demirkaya et al. [65]	0.85 open bite group 0.78 deep bite group	1.01 * open bite group 0.79 deep bite group	0.54 open bite group 0.68 deep bite group	downward and forward
Barrera-Mora et al. [63]	n/a	n/a	0.26 normal overjet 0.4 anterior crossbite * 0.15 increased overjet *	n/a
Utt et al. [51]	0.59/0.59 Angle class I right/left 0.63/0.64 Angle class II right/left 0.62/0.62 Angle class II/1 right/left 0.7/0.75 Angle class II/2 right/left	0.75/0.75 Angle class I right/left 0.91/0.88 Angle class II right/left 0.87/0.85 Angle class II/1 right/left 1.16/1.10 Angle class II/2 right/left	0.26 Angle class I 0.27 Angle class II 0.27 Angle class II/1 0.25 Angle class II/2	posteroinferior
He et al. [58]	1.29 left 1.25 right	1.59 left 1.56 right *	0.59	posterior and inferior
Turasi et al. [66]	0.97 large overjet group 0.73 control group	0.97 * large overjet group 0.64 control group	0.86 * large overjet group 0.52 control group	downward and backward

* statistically significant. delta x = condylar displacement in anteroposterior axis; delta z = condylar displacement in vertical axis; delta y = condylar displacement in transverse axis.

**Table 5:** Number of condyles with extreme displacement.

Author	Number of Joints with Extreme Horizontal Displacement above or below 2 mm	Number of Joints with Extreme Vertical Displacement above 2 mm	Number of Joints with Extreme Transverse Displacement above 0.5 mm
Ponces et al. [14]	14 (6.49%) Hyperdivergent group: 3 (4.17%) -posterior: 2 (2.78%) -anterior: 1 (1.39%) Hypodivergent group: 6 (8.33%) -posterior: 0 -anterior: 6 (8.33%) Intermediate group: 5 (6.9 4%) -posterior: 3 (4.17%) -anterior: 2 (2.78%)	60 (27.77%) Hyperdivergent 25 (34.72%) Hypodivergent 17 (23.61%) Intermediate 18 (25%)	n/a
Chandra et al. [61]	18 (12.86%) Hyperdivergent group: 15 (10.71%) Hypodivergent group: 3 (2.14%)	21 (15%) Hyperdivergent group: 15 (21.43%) Hypodivergent group: 6 (8.57%)	n/a
Girardot [62]	20/66 (30.3%) Hyperdivergent group: 16/33 (48.48%) -posterior: 10/33 (30.3%) -anterior: 6/33 (18.18%) Hypodivergent group: 4/33 (12.12%) -posterior: 3/33 (9.09%) -anterior: 1/33 (3.03%)	24/66 (36.36%) Hyperdivergent: 19/33 (57.57%) Hypodivergent: 5/33 (15.15%)	n/a
Radej et al. [64]	1 (1%)	6 (6.3%)	6 (6.3%)
Hidaka et al. [49]	1 (0.7%) left side 2 (1.3%) right side	14 (9.3%) left side 11 (7.3%) right side	47 (31.3%)
Barrera-Mora et al. [63]	n/a	n/a	n/a
Utt et al. [51]	5 (16.1%) Angle class I 15 (20.8%) Angle class II	n/a	17 (15.9%) 4 (12.9%) Angle class I 7 (11.3%) Angle class II
He et al. [58]	18% left side 17% right side	31% left side 28% right side	48%

**Table 6:** Number of condyles with displacement > 1 mm.

Author	Number of Joints with Vertical CR-CO Slide >1 mm	Number of Joints with Anteroposterior CR-CO Slide > 1 mm	Number of Joints with Transverse CR-CO slide > 1 mm
Ari-Demirkaya et al. [65]	Open bite group: 50% * Deep bite group: 27% Control group: 17%	No difference between groups	Open bite group: 13% Deep bite group: 33% Control group: 13%
Turasi et al. [66]	39% overjet group 18% control group	39% overjet group 24% control group	33% overjet group 15% control group

* statistically significant.

## Data Availability

Not applicable.

## Appendix A

Search strategy and terms used for the search.

**Database and Limits**	**Key Words**		
	**Semantic Fields: “Maxillofacial Structure Or Malocclusion”**		**Semantic Field: “Condylar Displacement”**
PubMed (n = 897)	orthodontic OR cephalometr* OR sagittal skeletal OR sagittal relationship OR skeletal relationship OR skeletal pattern OR facial pattern OR vertical pattern OR vertical relationship OR “anb angle” OR horizontal relationship OR dentofacial deformit* OR dentofacial orthop* OR maxillofacial structure OR malocclusion OR occlusal relationship OR “midline shift” OR development, maxillofacial[MeSH Terms] OR abnormalities, maxillofacial[MeSH Terms] OR dental occlusion[MeSH Terms])	**AND**	centric relation intercusp* OR centric slide OR condylar displacement OR centric discrepancy
Web of Science (n = 604)	orthodontic OR cephalometr* OR “sagittal skeletal” OR “sagittal relationship” OR “skeletal relationship” OR “skeletal pattern” OR “facial pattern” OR “vertical pattern” OR “vertical relationship” OR “anb angle” OR “horizontal relationship” OR “dentofacial deformit*” OR “dentofacial orthop*” OR “maxillofacial structure” OR “malocclusion” OR “occlusal relationship” OR “midline shift” OR “maxillofacial development” OR “maxillofacial abnormalities” OR “dental occlusion”	**AND**	centric relation intercusp* OR centric slide OR condylar displacement OR centric discrepancy
Scopus (n = 779)	orthodontic OR cephalometr* OR sagittal skeletal OR sagittal relationship OR skeletal relationship OR skeletal pattern OR facial pattern OR vertical pattern OR vertical relationship OR “anb angle” OR horizontal relationship OR dentofacial deformit* OR dentofacial orthop* OR maxillofacial structure OR malocclusion OR occlusal relationship OR “midline shift” OR maxillofacial development OR maxillofacial abnormalities OR dental occlusion	**AND**	centric relation intercusp* OR centric slide OR condylar displacement OR centric discrepancy
The Cochrane Library (n = 40)	orthodontic OR cephalometr* OR sagittal skeletal OR sagittal relationship OR skeletal relationship OR skeletal pattern OR facial pattern OR vertical pattern OR vertical relationship OR “anb angle” OR horizontal relationship OR dentofacial deformit* OR dentofacial orthop* OR maxillofacial structure OR malocclusion OR occlusal relationship OR “midline shift” OR development, maxilofacial [MeSH Terms]. OR abnormalities, maxillofacial[MeSH Terms]. OR dental occlusion[MeSH Terms])		Centric relation-maximum intercuspation discrepancy OR Centric Relation-Centric Occlusion discrepancy OR Centric Slide OR centric discrepancy
